# Enhancing the confidence of potential targets enriched by similarity-centric models: the crucial role of the similarity threshold

**DOI:** 10.3389/fphar.2025.1574540

**Published:** 2025-08-08

**Authors:** Ling-Fei Tong, You-Jin Ge, Su-Qing Yang

**Affiliations:** ^1^ Department of Pharmacy, Jiangxi Provincial People’s Hospital, The First Affiliated Hospital of Nanchang Medical College, Nanchang, Jiangxi, China; ^2^ Office of Drug Clinical Trials Institution, Nanchang People’s Hospital (The Third Hospital of Nanchang), Nanchang, Jiangxi, China

**Keywords:** target prediction, drug–target interactions, polypharmacology, drug repositioning, adverse effects, similarity threshold

## Abstract

**Background:**

Computational target fishing (TF) tools have made tremendous progress in narrowing down the set of potential targets, thereby expediting time- and resource-consuming wet-lab experiments. Among these tools, similarity-centric TF methods are particularly prominent and extensively employed to guide target identification in modern research. Despite substantial progress, similarity-centric models still have significant limitations, particularly regarding the confidence of enriched targets.

**Methods:**

We constructed several baseline similarity-based TF models to explore supplementary aspects that could enhance the confidence of enriched targets. A high-quality library was first constructed. Multiple fingerprint representations and scoring schemes were applied to construct individual or ensemble models. The leave-one-out-like cross-validation and rigorous validation metrics were used to measure the performance. Based on the performance under different conditions, multiple influential factors, focusing on the similarity threshold, were investigated.

**Results:**

Evidence showed that the similarity between the query molecule and the reference ligands that bind to the target could serve as a quantitative measure of the target reliability. The distribution of effective similarity scores for TF was fingerprint-dependent. To highlight the identification of true positives by filtering background noise and to maximize reliability by balancing precision and recall, the corresponding similarity thresholds for each fingerprint type were identified. Furthermore, additional influential factors, including the choice of different fingerprints, the integration of different models, the target-ligand interaction profile, and the promiscuity of the query molecule, were investigated.

**Conclusion:**

Collectively, our findings provide novel insights into enhancing the confidence of enriched targets by applying the similarity threshold and other perspectives. These results also lay the groundwork for developing more robust and reliable target prediction models in the future.

## Graphical Abstract

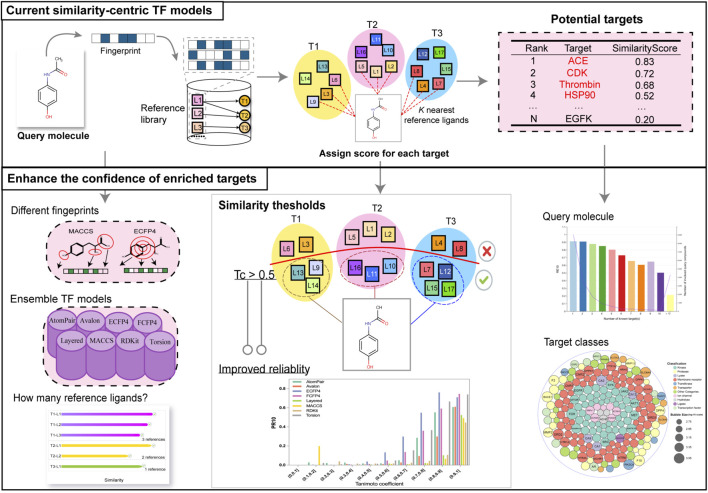

## 1 Introduction

In the early stage of drug discovery, abundant bioactive candidates have been identified, ranging from natural products isolated from natural resources (Luo et al., 2019; Chen et al., 2023) to experimentally synthesized small molecules (Rankin and Poulsen, 2017) and bioactive compounds screened by high-throughput screening (Mayoh et al., 2023; Khambhati et al., 2024) or cell-based phenotypic screening (Moffat et al., 2014; Selvin et al., 2023). However, the targets of the vast majority of known chemical compounds have not been completely clarified, which is a major obstacle in their utilization (Zhao et al., 2023).

Target identification is vital for rationalizing the bioactivities of small molecules, providing structural optimization to improve efficacy, and indicating possible side effects. It is estimated that 52% of clinical phase-II failures are primarily attributed to insufficient efficacy (Harrison, 2016), among which, most are caused by poor targeting (Jorgensen, 2009; El-Wakil et al., 2017) or unfavorable off-target effects (Park et al., 2024; Song et al., 2020). The identification of potential targets for drug candidates in advance may discover potential adverse effects, thereby reducing the attrition rate in clinical trials. Moreover, it is well-known that most drugs bind to multiple targets, a general phenomenon known as “polypharmacology” (Kabir and Muth, 2022; Ryszkiewicz et al., 2023). The interactions between the secondary targets and drugs may offer opportunities for drug repurposing, as exemplified by the well-known case of sildenafil (Ashburn and Thor, 2004; Jourdan et al., 2020; Houslay, 2016; Duan et al., 2024).

The traditional experimental approaches for target identification are typically time- and labor-consuming, making target identification inefficient (Rix and Superti-Furga, 2009; Chen et al., 2017; Lee and Bogyo, 2013; Drewes and Knapp, 2018). In the past few years, open bioactivity data accumulated in public data repositories such as ChEMBL (Zdrazil et al., 2024), BindingDB (Gilson et al., 2016), and PubChem BioAssay (Wang et al., 2017) databases have grown tremendously in size, which enables us to narrow down potential target candidates to a small set by automatically screening chemical compounds against a bioactivity database. It is highly beneficial as focusing experimental confirmatory tests on the most reliable predictions will lead to much higher hit rates.

Among target prediction (also called target fishing, TF) methods, similarity-centric approaches have made tremendous progress due to their flexibility, relatively low computational cost, and remarkable predictive performance (Cereto-Massagué et al., 2015; Rollinger et al., 2009; Yang et al., 2023; Agamah et al., 2020). Through screening a query molecule (i.e., the molecule to be predicted) against a huge bioactivity database (i.e., a reference library), each known target is quantified by similarity scores calculated between the query molecule and its K closest reference ligands of each target. Targets with higher scores are then identified as potential candidates. State-of-the-art similarity-based TF tools are as follows: SwissTargetPrediction (Daina et al., 2019), the Polypharmacology Browser (PPB) (Awale and Reymond, 2017), the Polypharmacology Browser 2 (PPB2) (Awale and Reymond, 2019), TargetHunter (Wang et al., 2013), MuSSeL (Alberga et al., 2019), ChemMapper (Gong et al., 2013), HitPickV2 (Hamad et al., 2019), TarPred (Liu et al., 2015), MolTarPred (Peon et al., 2019), and others (Liu et al., 2014; AbdulHameed et al., 2012).

However, the confidence levels of enriched targets provided by these tools are typically limited to the ranking order of the potential targets, which is insufficient for researchers to make thoughtful decisions. In practice, when employing similarity-centric methods for TF, the similarity between the query molecule and the reference ligands that bind to the potential target can serve as a crucial indicator of confidence. Furthermore, the performance of the TF models can be improved by applying the similarity thresholds to filter out background noise (i.e., the intrinsic similarities between two random molecules), thereby improving the confidence of the hit targets. Additionally, several other factors, such as the choice of different fingerprints and the integration of different models, can also influence the performance of the TF models.

In this study, we constructed several baseline similarity-based TF models to explore several aspects that can enhance the confidence of predictions, with a particular focus on the similarity threshold. To lay a solid foundation, a high-quality dataset was constructed as the reference library. Several baseline models were developed using different scoring schemes and various fingerprints. To simulate actual TF scenarios, a leave-one-out-like cross-validation was performed, and rigorous validation metrics were designed to comprehensively assess the performance of these models. Based on the optimal scoring schemes, the relationship between the similarity scores and the prediction reliability was assessed. Following that, fingerprint-specific similarity thresholds to retrieve true positives and maximize the identification by balancing recall and precision were determined. Furthermore, additional informative aspects expected to enhance prediction confidence, including the choice of different fingerprints and the integration of different models, were investigated. Finally, other influential factors, such as target–ligand interaction profiles and the promiscuity of the query molecule, were explored to provide a comprehensive understanding of confidence measurement in TF predictions.

## 2 Materials and methods

### 2.1 Preparation of the reference library

A total of 1,460 human protein targets were collected from the ChEMBL v34 database (Zdrazil et al., 2024) for TF. Then, ligands associated with these targets, together with their corresponding bioactivity data (including IC_50_, K_i_, K_d_, or EC_50_), were retrieved from the BindingDB (Gilson et al., 2016) and ChEMBL v34 databases (Zdrazil et al., 2024). Due to the integration of different sources, multiple bioactivity data may be found for one ligand–target pair. For such ligand–target interactions, the pair was retained only if all of the corresponding bioactivity values differed by no more than an order of magnitude, and the median value was used as the definitive activity for that pair (Yang et al., 2023). To ensure the high quality of the reference library, the ligand–target pairs with strong bioactivity (IC_50_, K_i_, K_d_, or EC_50_ < 1 μM) were maintained.

**FIGURE 1:**
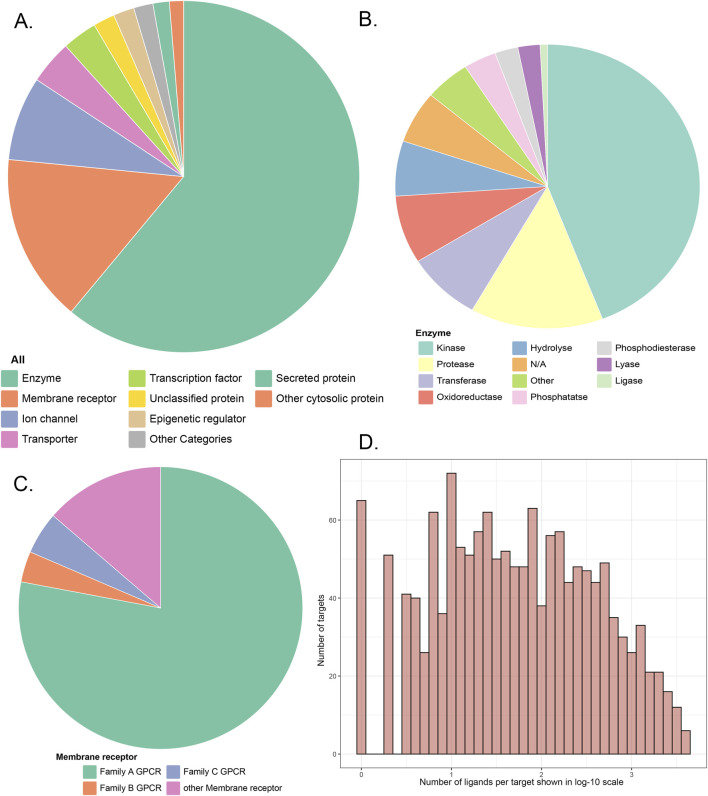
**(A)** Category distribution of targets that can be predicted in our similarity-centric TF models; **(B,C)** subgraphs of A; **(D)** distribution of the number of reference ligands against each target in the reference library.

The entire reference library contains 1,460 proteins, 278,583 ligands, and 406,289 ligand–target interactions. The targets have been approved by the FDA or proven to be therapeutic, which mainly includes enzymes, membrane receptors, ion channels, transporters, and proteins from other detailed categories (Figures 1A–C). Among the 1,460 targets, 75.7% have more than 10 ligands, 565 of them have more than 100 ligands, and 124 targets are associated with more than 1,000 ligands (Figure 1D).

### 2.2 Construction of the similarity-centric TF models

For TF, two-dimensional fingerprints are extensively employed to characterize chemical structures, without considering the spatial coordinates of molecular atoms (Trosset and Cavé, 2019). In our study, the RDKit (http://www.rdkit.org/) package (Landrum, 2013) was utilized to compute eight distinct fingerprints for each compound, including AtomPair (Carhart et al., 1985), Avalon (Gedeck et al., 2006), ECFP4 (Capecchi et al., 2020), FCFP4 (Sawada et al., 2014), RDKit (Zhang et al., 2024), Layered (Landrum, 2013), MACCS (Muchmore et al., 2008), and Torsion (Nilakantan et al., 1987) fingerprints, as detailed in Table 1. Each of the molecular fingerprints has its unique characteristics, offering a diverse range of perspectives for investigating the relationship between molecular structures and potential targets.

**TABLE 1:** Fingerprints employed for TF in our study.

Fingerprint	Description	Number of bits
AtomPair	A fingerprint encoding molecular shape based on the distance and type between pairs of atoms, which is often used for scaffold-hopping (Carhart et al., 1985).	1,024
Avalon	A fingerprint based on hashing algorithms, which provides a rich molecular description by generating larger bit vectors that enumerate certain paths and feature classes of the molecular graph (Gedeck et al., 2006).	1,024
ECFP4	The atom-centered circular fingerprint ECFPs (extended-connectivity fingerprints) with diameter = 4, which belongs to the best-performing fingerprints in small molecule virtual screening (Capecchi et al., 2020).	1,024
FCFP4	The atom-centered circular fingerprint FCFPs (functional-class fingerprints) with diameter = 4, which describes functional roles of the atoms and thus involves a smaller set of features than ECFP (Sawada et al., 2014).	1,024
RDKit	A fingerprint based on the substructures and chemical features of the molecule, which is suitable for rapid screening and comparison of molecular similarity (Zhang et al., 2024).	1,024
Layered	Substructure-matching fingerprints (Landrum, 2013).	1,024
MACCS	A fingerprint recording the occurrence of 166 predefined chemical substructures, which is commonly used for compound database searches and molecular similarity comparisons (Muchmore et al., 2008).	166
Torsion	A fingerprint based on the molecular topology, such as the arrangement of rings and bonds, which is suitable for describing the overall architecture and topological characteristics of a molecule (Nilakantan et al., 1987).	1,024

Given a query compound, pairwise fingerprint-based similarity searching runs through the entire reference library, where the similarity between the query compound and each of the reference ligands is measured by the Tanimoto coefficient (Tc) (Bajusz et al., 2015; Dunn et al., 2023). For a given target represented by its associated reference ligands, its score as a potential target is quantified by the similarities of a predefined number of *K* most similar reference ligands to the query compound. The potential targets are marked as a list of top-ranked predictions in a descending order according to three scoring schemes: (1) *K*NNTc: the average similarity of the *K* most similar ligands of a target to the query compound; (2) MaxTc: it is a special case of *K*NN when *K* equals to 1, considering only the most similar ligand against the query compound; (3) MeanTc: the average similarity between the query molecule and all ligands associated with a target.

### 2.3 Ensemble similarity-centric TF models

In our study, two types of ensemble TF methods were implemented, defined as the similarity ensemble model and the rank order ensemble model, aimed to leverage the strengths of multiple fingerprints and improve the overall performance of target prediction. For the former one, due to different similarity coverage intervals across fingerprints, scores obtained from one fingerprint for each target were standardized using the formula $Z_{score}=\left(x-µ\right)/\sigma$ , where x is the original score of the target, and µ and σ are the average score and the variance score spanned from 1,460 targets, respectively. After the standardization, the scores from different fingerprints can be ensembled. For each of the 247 (2^8^–9) ensemble combinations, the score of each target is redistributed as ${\text{Score}}_{1}=\sum_{i=2}^{m}Z_{score}$ , where m is the number of fingerprints in the combinations and $Z_{score}$ is the standardization scores outputted by different fingerprints in the combinations. For the latter one, for each of the 247 ensemble combinations, the score of each target is redistributed as ${\text{Score}}_{2}=\sum_{i=2}^{m}rank$ , where m is the number of fingerprints in the combinations, and the rank is the rank order outputted by fingerprints in the combinations.

### 2.4 Performance evaluation

To simulate real-world target prediction events, the leave-one-out cross-validation (CV) was typically employed to measure the performance of TF models. Given the substantial computational resources required for traditional leave-one-out CV, a modified leave-one-out-like CV was performed. In the procedure, representative compounds with distinct Murcko scaffolds were extracted from the reference library. Each of the representatives was sequentially taken as the query compound, while the remaining compounds in the library after excluding the query molecule were taken as the reference ligands for TF. Compared with the traditional n-fold CV, the leave-one-out-like CV procedure ensures that the reference library is integral and that the targets to be validated are distributed across almost all targets, thereby providing a more realistic and unbiased assessment of TF performance.

**FIGURE 2:**
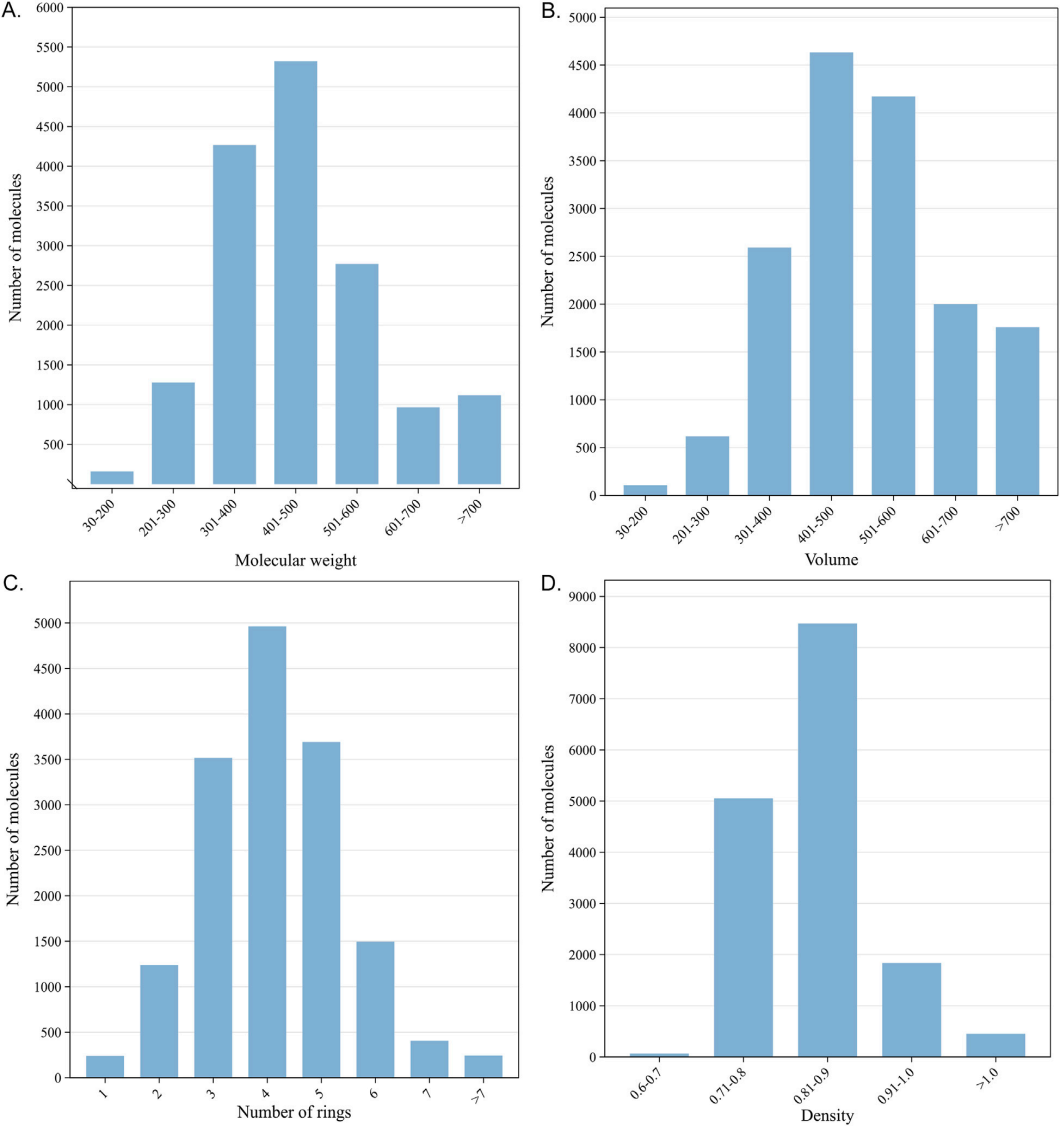
Physicochemical properties of 15,876 validation molecules: **(A)** molecular weight; **(B)** volumes; **(C)** number of rings; **(D)** density.

The Murcko scaffolds of 278,583 reference ligands were calculated using MOE software v 2018. A total of 96,817 unique Murcko scaffolds were identified, with an average of 71.6 scaffolds per target. To construct a representative dataset for evaluation, a stratified sampling strategy based on the number of Murcko scaffolds associated with each target was employed. Specifically, for targets with fewer than 70 scaffolds, 30% of the ligands with distinct scaffolds were randomly selected. For targets with 70 or more scaffolds, 20 ligands with distinct scaffolds were randomly extracted. This approach ensured a diverse representation of scaffolds across different targets. Finally, a subset of 15,876 ligands, 1,348 protein targets, and 21,177 pairs of protein–ligand interactions were selected for evaluation, representing 21,177 TF events. The physicochemical properties of these validation molecules are illustrated in Figure 2.

To evaluate the performance of the above TF models, we used three key metrics, namely, precision (PR_n_), recall (RE_n_), and F_1_ score. These metrics are defined as follows:

1. Recall (RE_n_): This metric represents the fraction of known targets that are correctly identified (predicted as positive) out of all known targets. It is widely used to measure the TF performance. It is calculated as follows:

$${\text{RE}}_{n}=\frac{\text{TPn}}{\text{TPn}+\text{FNn}}$$

where FN_n_ is the number of false negative predictions (known targets not predicted as known) among the top-ranked n targets.

2. Precision (PR_n_): This is the fraction of known targets that are correctly predicted among the top-ranked n targets. It is calculated as follows:

$${\mathrm{PR}}_{n}=\frac{\text{TPn}}{\text{TPn}+\text{FPn}}$$

where TP_n_ is the number of true positive predictions (known targets correctly predicted) in the top-ranked n targets and FP_n_ is the number of false positive predictions (non-known targets predicted as known) in the top-ranked n targets.

3. F_1_ score: This is the harmonic mean of precision and recall, and it provides a balanced measure of the two. A higher F_1_ score means a better performance in discriminating known targets based on an overall consideration. The F_1_ score is calculated as follows:

$$F_{1}=\frac{2*\text{REn}*\text{PRn}}{\text{PRn}+\text{REn}}$$

## 3 Results

### 3.1 The performance of models based on different scoring schemes

**FIGURE 3:**
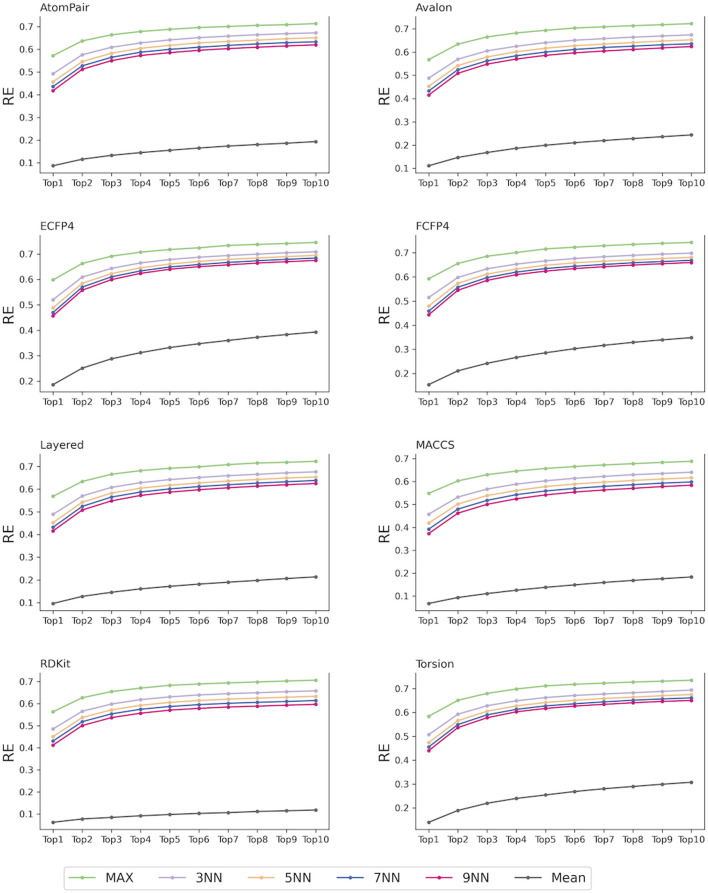
TF performance of the similarity-centric models based on different scoring schemes.

In our study, 40 target prediction models were constructed based on eight fingerprints and five scoring schemes. For each fingerprint, the strategies of considering the 1, 3, 5, 7, and 9 most similar ligands to the query compounds and all ligands of each target as the reference compounds for TF were designated as MaxTc, 3NNTc, 5NNTc, 7NNTc, 9NNTc, and MeanTc scoring schemes, respectively. As shown in Figure 3, models that considered several nearest reference ligands (e.g., MaxTc and *K*NNTc scores) outperformed the model that included all the reference ligands (e.g., MeanTc). Specifically, for eight fingerprints, the recall of the top-ranked single targets (RE_1_) of the MaxTc, 3NNTc, 5NNTc, 7NNTc, and 9NNTc scoring schemes exceeded 0.35, while the RE_1_ of the MeanTc scoring strategy was less than 0.20. The recall of the top-ranked ten targets (RE_10_) of the MaxTc, 3NNTc, 5NNTc, 7NNTc, and 9NNTc scoring schemes surpassed 0.58, while the RE_10_ of the MeanTc scoring strategy remained below 0.39. The results indicate the powerful performance of the constructed similarity-centric TF models. Additionally, the findings illustrated that the reference ligands with high structural similarity to the query compounds may capture more specific molecular information for each target and better distinguish it from others.

For each of the fingerprints, the MaxTc scoring scheme demonstrated the best performance, with RE_1_ and RE_10_ exceeding 0.5 and 0.65, respectively. This was aligned with several state-of-the-art similarity-centric TF tools: SwissTargetPrediction (Daina et al., 2019), the Polypharmacology Browser (PPB) (Awale and Reymond, 2017), Polypharmacology Browser 2 (PPB2) (Awale and Reymond, 2019), TargetHunter (Wang et al., 2013), MuSSeL (Alberga et al., 2019), etc (Liu et al., 2015; Peon et al., 2019). In the following studies, given the superior performance demonstrated by the MaxTc scoring scheme, potential targets will be scored using the nearest reference ligand of each target to the query compounds.

### 3.2 Fine fingerprints outperform sketchy fingerprints

**FIGURE 4:**
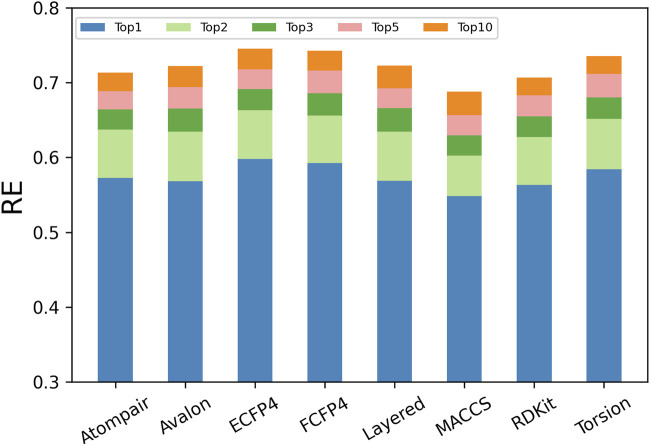
Performance of models in TF when applying different types of fingerprints.

The descriptors used to represent compounds decide the application range and success of a prediction model. Structural descriptions at different levels sketch different aspects of compound behaviors and provide diverse clues for inferring potential targets. It was further explored which fingerprints performed better based on the same scoring strategy (i.e., MaxTc) for TF. As shown in Figure 4, the RE_n_ increased with the increase in *n* (the number of top-ranked predictions considered), and the increasing trend gradually slowed down for each fingerprint.

However, the performance of the eight types of fingerprints varied significantly, where RE_1_ ranged from 0.54 to 0.59, RE_5_ ranged from 0.65 to 0.72, and RE_10_ ranged from 0.67 to 0.75. Among the eight fingerprints, the circular fingerprints (ECFP4 and FCFP4) performed the best, and they predicted more than 59%, 71%, and 74% of known targets at the top-1, top-5, and top-10 of the prediction list, respectively. Fingerprints based on molecular fragments/substructures (RDKit, Avalon, and Layered) and molecular topology/shape (AtomPair and Torsion) performed moderately, with the RE_1_, RE_5_, and RE_10_ being greater than 0.56, 0.68, and 0.71, respectively. The fingerprint based on a small number of predefined molecular fragments (MACCS) performed the worst, with the RE_1_, RE_5_, and RE_10_ being equal to 0.55, 0.66, and 0.69, respectively.

The ECFP4 fingerprint outperformed all other fingerprints, with 59.82%, 71.61%, and 74.53% of known targets found in the top-1, top-5, and top-10 of the prediction list, respectively. Given that predictions were made among the 1,460 human targets, the RE_1_ and RE_10_ corresponded to approximately 873-fold (59.82%/ (1/1,460)) and 109-fold (74.53%/ (10/1,460)) enrichment compared to that with random picking, respectively. Our results were consistent with the fact that the ECFP4 fingerprint has been widely used for TF (Awale and Reymond, 2019).

### 3.3 The confidence of hit targets will be improved by applying the similarity thresholds

**FIGURE 5:**
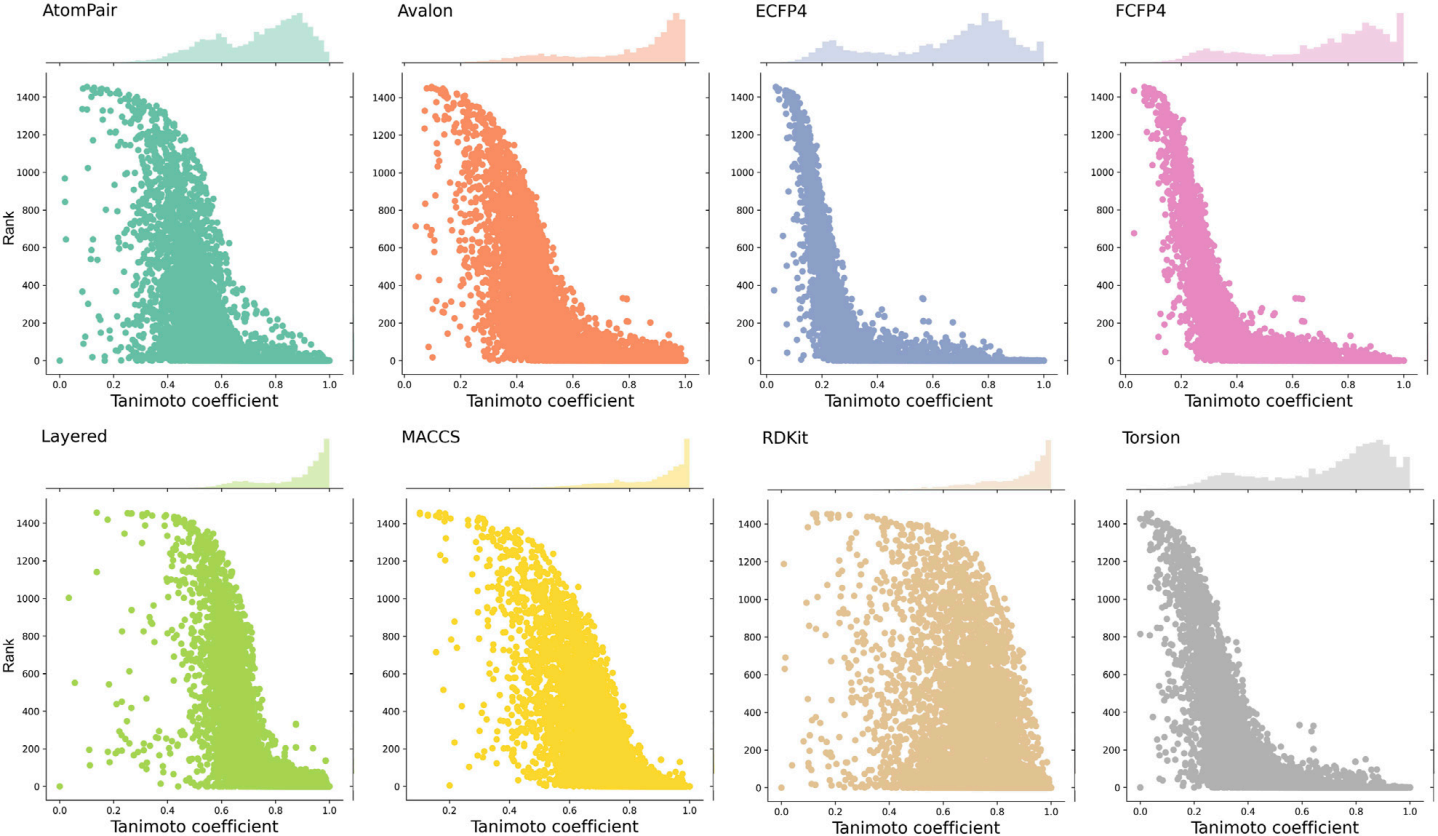
Distributions between the similarity scores and the rank order of known target(s). Each point on the scatter plot represents a known target. The x-axis represents the similarity score (MaxTc) between the query compound and the reference ligand of its known target(s). The y-axis refers to the rank order of the known target(s) in the prediction list.

Given that each fingerprint captures distinct molecular fragments or properties, the same pair of compounds can exhibit varying similarity scores depending on the fingerprint employed. Observing the similarity baselines of different fingerprints helps us understand that the similarity ranges of different fingerprints for enriching active targets are diverse. Here, we calculated the similarity (namely, MaxTc) between each of the query compounds and its nearest reference ligand associated with each of its known targets and then plotted a scatter plot to depict the distribution between the similarity and the rank order of the known target in the prediction list. Although the predictive performance of the eight fingerprints was comparable, the similarity distributions varied with fingerprints, as visualized in Figure 5.

ECFP4, FCFP4, and Torsion fingerprints yield a broad range of similarity scores, spanning from 0.2 to 1. The similarity scores obtained by AtomPair and Avalon fingerprints enriched from 0.4 to 1.0. RDKit, Layered, and MACCS fingerprints produce a narrow distribution, predominantly concentrated in the range of 0.6–1.0. This can be attributed to the varying levels of structural detail captured by each fingerprint type. For fingerprints with more comprehensive structural information (e.g., ECFP4 and FCFP4), a smaller variation in the similarity score was required to reflect structural alterations, while for fingerprints that capture less detailed structural representation (e.g., MACCS), a wider range of the scores was needed to cover the same degree of changes.

To highlight the identification of true positives by filtering the background noise, namely, the intrinsic similarities between two random molecules, the TF performance was evaluated across different similarity cutoffs. Fingerprint-specific similarity thresholds were subsequently determined. As researchers typically select the top-10 predictions for further experimental investigation, the metric of precision at rank 10 (PR_10_) was used to explore the similarity threshold for true positive predictions among the ten candidates.

**FIGURE 6:**
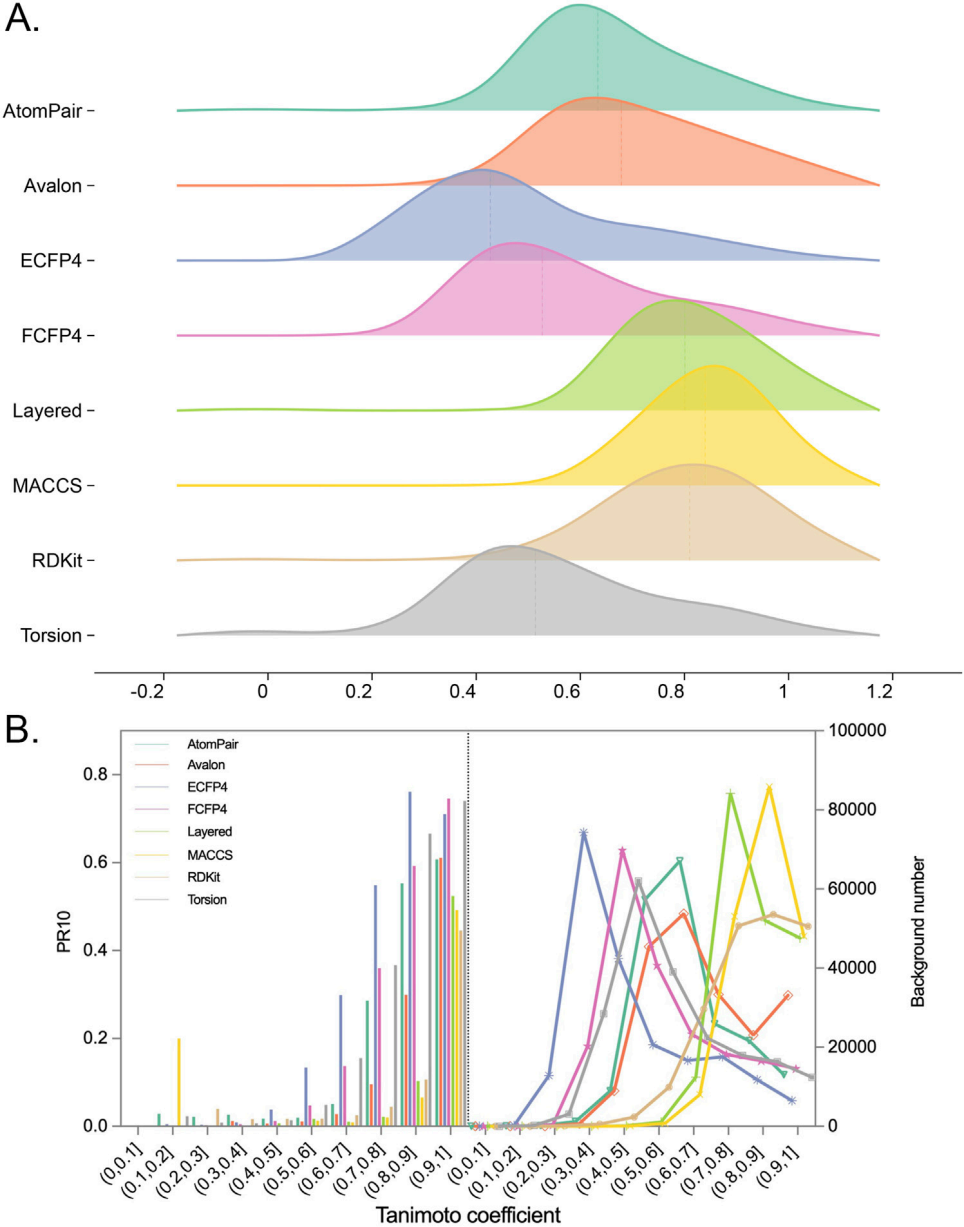
**(A)** Distributions of Tc between each query compound and the most similar ligand of its top-10 potential targets for each fingerprint. **(B)** Plot depicting the relationship between Tc ranges and PR_10_. The ranges of Tc were split into 10 bins. In each bin, the PR_10_ or the number of background targets (namely, the top-10 targets for all query compounds) were calculated separately for query compounds with Tc falling into this bin.

Figure 6A shows the distributions of the MaxTc scores between each query compound and its nearest reference ligand associated with each of the top-10 targets in the prediction list. The results of setting similarity thresholds demonstrated that for each fingerprint, as the similarity threshold increased, PR_10_ gradually increased, as shown in Figure 6B. For example, PR_10_ of the FCFP4 fingerprint increased from 0 to 0.75, while that of the AtomPair fingerprint increased from 0 to 0.61. The MACCS fingerprint, which recorded 166 substructure fragments that may only reflect a slight change in similarity scores for the alteration in key groups between two molecules, showed an increased PR_10_ from 0 to 0.49.

Importantly, the upward trends of PR_10_ varied across fingerprints, indicating that the similarity thresholds required to retrieve true positive targets (namely, known ligand–target interactions) differed among them. For example, the PR_10_ of the ECFP4 fingerprint improved when the similarity exceeded 0.4, suggesting that when the similarity between the query molecule and the reference ligand was between 0 and 0.4, the probability of the prediction becoming the real target was nearly 0. The similarity thresholds for obtaining true positives of ECFP4, FCFP4, Torsion, AtomPair, Avalon, RDKit, Layered, and MACCS fingerprints were determined to be 0.4, 0.5, 0.5, 0.6, 0.6, 0.7, 0.7, and 0.7, respectively. The “TP Similarity Threshold” column in Table 2 presents the threshold information for each fingerprint. The improved enrichment rate shown in the table directly indicates that the confidence of the enriched targets can be improved by applying the similarity thresholds. The finding shows that if the similarity is below a predefined threshold, the target, regardless of its rank order, cannot be considered a potential hit.

**TABLE 2:** True-positive similarity thresholds for each fingerprint.

Fingerprint	TP similarity threshold	Recall^$^	Precision^$^	Improved enrichment rate^&^
AtomPair	0.60	0.65	0.24	1.47
Avalon	0.60	0.67	0.22	1.36
ECFP4	0.40	0.69	0.28	1.69
FCFP4	0.50	0.69	0.27	1.66
RDKit	0.70	0.64	0.20	1.24
Layered	0.70	0.68	0.17	1.08
MACCS	0.70	0.64	0.16	1.04
Torsion	0.50	0.67	0.29	1.78

^$^Metrics obtained by applying different similarity thresholds.

^&^Fold increase in the efficiency of target identification, as measured by the reduction in the total number of targets that need to be picked (i.e., background targets as described in Figure 6B) to obtain the same recall rate, compared to the scenario where no similarity threshold is applied.

### 3.4 Optimal similarity thresholds to balance the trade-off between precision and recall

**FIGURE 7:**
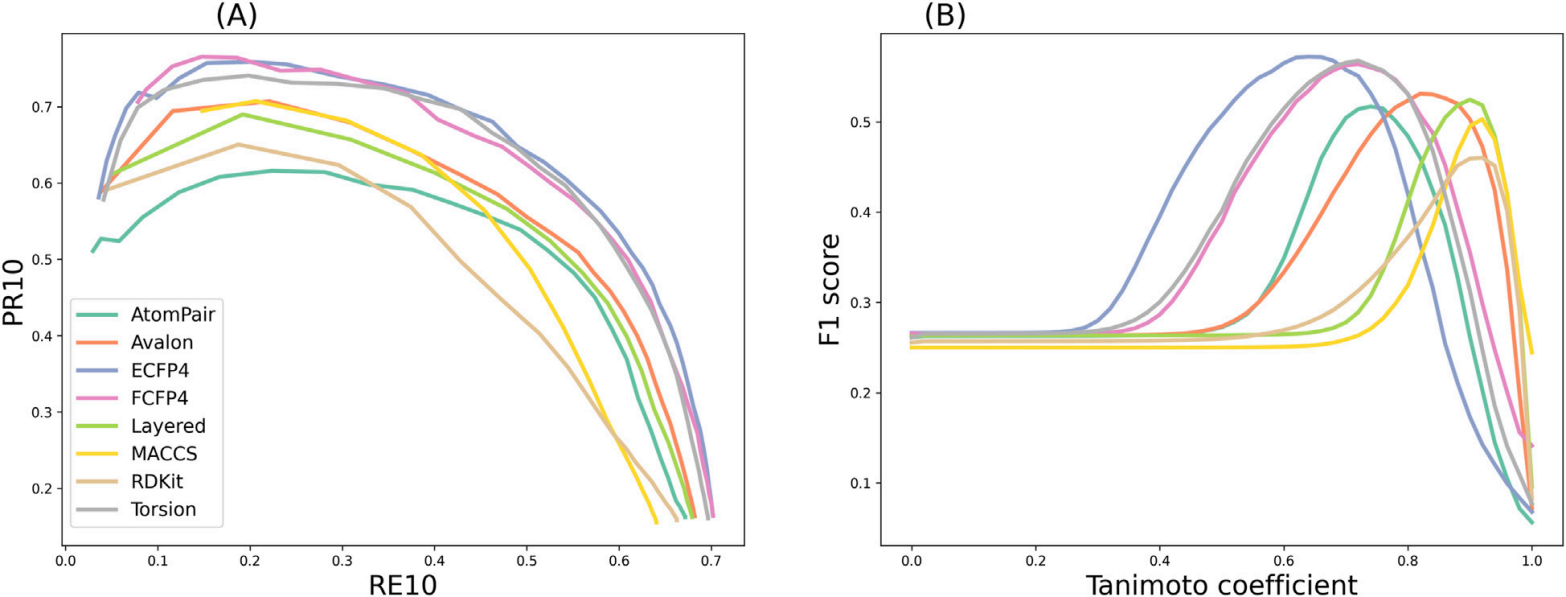
**(A)** Precision–recall curves, where the data of PR_10_ and RE_10_ in each point were obtained by setting different similarity cutoffs from 0 to 1. **(B)** F_1_ scores obtained by applying the similarity cutoffs with the step size of 0.01 for TF.

Despite the increase in PR_10_ (having fewer false positives) with the increase in similarity cutoffs, it comes at the expense of RE_10_ (true positives). In other words, as stricter thresholds were used to provide predictions, the prediction coverages were sacrificed. The trade-off between PR_10_ and RE_10_ was most visible in precision–recall curves by setting different similarity cutoffs, as shown in Figure 7A. Therefore, a compromise was required between the ability to provide large-scale predictions and the retrieval of actual targets. The F_1_ score is the harmonic mean of precision and recall, and it provides a balanced measure of the two.

As shown in Figure 7B, the results indicated that the F_1_ curves of the eight fingerprints that provide the best compromise between the two conflicting objectives were diverse. The maximum F_1_ score of the ECFP4 fingerprint was as high as 0.57, which could be obtained at a similarity of 0.64. The maximum F_1_ score of the MACCS fingerprint was as high as 0.50, which could be obtained at a similarity of 0.92. The threshold information of the remaining fingerprints is shown in the “Balanced Similarity Threshold” column in Table 3. This finding demonstrates that the modulation of the similarity threshold can enhance the confidence of models to discriminate known targets based on an overall consideration. It provides researchers with concrete evidence for selecting appropriate similarity thresholds to effectively balance the trade-off between sensitivity and specificity in target prediction, which is valuable for several TF tools, such as SwissTargetPrediction, PPB2, PPB, HitPickV2, TargetHunter, MolTarPred, and TarPred.

**TABLE 3:** Balanced similarity thresholds for each fingerprint.

Fingerprint	Balanced similarity threshold	Recall^$^	Precision^$^	F_1_^$^	Improved enrichment rate^&^
AtomPair	0.74	0.52	0.51	0.52	3.16
Avalon	0.82	0.56	0.51	0.53	3.12
ECFP4	0.64	0.56	0.58	0.57	3.54
FCFP4	0.70	0.57	0.55	0.56	3.37
RDKit	0.90	0.47	0.45	0.46	2.81
Layered	0.88	0.56	0.48	0.52	2.97
MACCS	0.92	0.45	0.56	0.50	3.63
Torsion	0.70	0.56	0.57	0.57	3.56

^$^Metrics obtained by applying different similarity thresholds.

^&^Fold increase in the efficiency of target identification, as measured by the reduction in the total number of targets that need to be picked (i.e., background targets as described in Figure 6B) to obtain the same recall rate, compared to the scenario where no similarity threshold is applied.

### 3.5 Ensemble models offer stable confidence for TF

Due to the different strengths of different fingerprints in TF applications, the confidence can also be enhanced by integrating similarity scores from different fingerprints or by using ensemble methods that weigh the contributions of each fingerprint based on its performance. Since one fingerprint may not contain enough features to fully characterize the chemical and biological spaces of the data, the occurrence of “activity cliff” was provided , which presents pairs of compounds with high structural similarity but unexpectedly large activity (or property) difference (Dunn et al., 2023). As a supplement, such a gap may be captured by other types of fingerprints.

Our study evaluated two ensemble models: the similarity ensemble method and the rank order ensemble method. For the similarity ensemble method, the average RE_1_, RE_2_, RE_3_, RE_5_, and RE_10_ were 55.12%, 63.45%, 67.20%, 70.27%, and 73.03%, respectively. For the rank order ensemble method, the average RE_1_, RE_2_, RE_3_, RE_5_, and RE_10_ were 54.17%, 62.06%, 65.65%, 68.54%, and 71.32%, respectively. Demonstrating superior predictive performance across evaluated ranks, the former ensemble model performed better than the latter one and, thus, was further analyzed.

**FIGURE 8:**
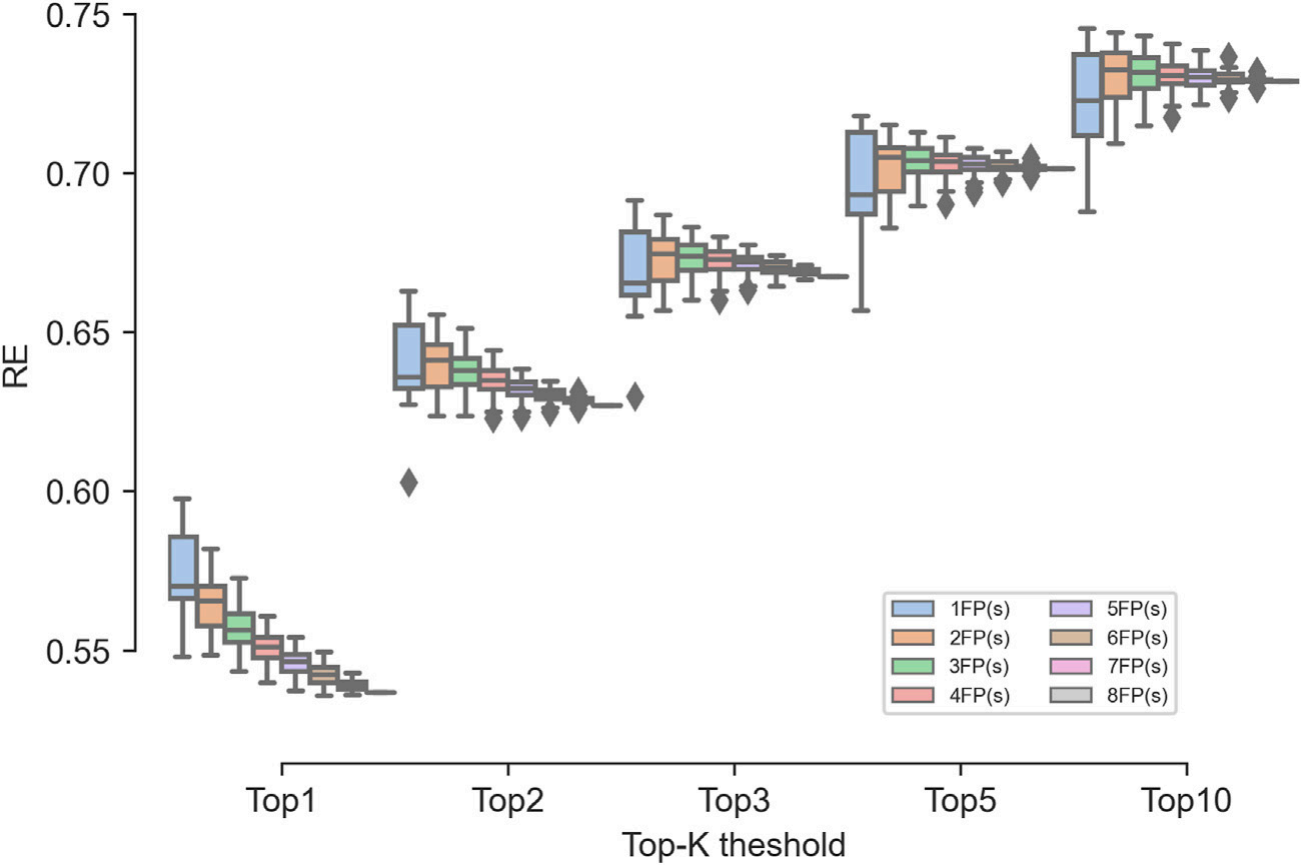
Effect of the number of fingerprints involved in the ensembled combinations on the TF performance.

As shown in Figure 8, the RE obtained by the single fingerprints varied greatly, while the RE obtained by the ensembled fingerprints varied slightly. For the RE_1_ metric, as the number of ensembled fingerprints increased, and the performance gradually deteriorated to even lower than the worst-performing MACCS fingerprint. For the RE_2_ to RE_10_ metrics, as the number of ensembled fingerprints increased, the average performance was equal to or superior than that of a single fingerprint. In addition, the RE of the ensembled fingerprints was much higher than that of the MACCS fingerprints and multiple single fingerprints, indicating the robust ability of the ensemble fingerprint to enrich known targets. Among the ensembled combinations, the combinations of two fingerprints showed the best performance, where the median RE was the highest among all fingerprints. Therefore, if a stable confidence is desired, the use of integrated fingerprints is valuable.

### 3.6 Other factors that assist in measuring the prediction confidence

Measuring the confidence of target predictions in similarity-centric models requires a multifaceted approach. While similarity scores and fingerprints form the foundation, incorporating additional factors, such as the target–ligand interaction profiles and the promiscuity of the query molecule, can significantly enhance the confidence and interpretability of predictions.

**FIGURE 9:**
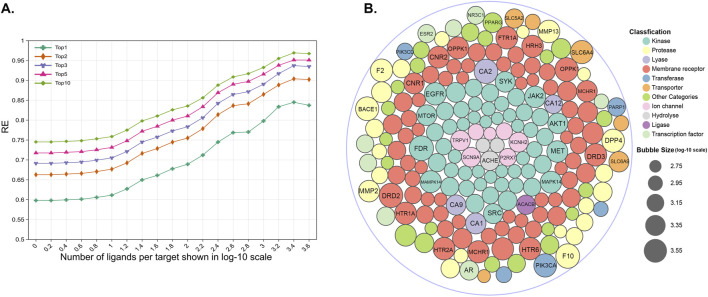
**(A)** RE for different targets with the increasing number of reference ligands. Each dot represents the calculated RE for all the test compounds with a known target number exceeding the x-axis number. **(B)** A part of targets with the greatest number of reference ligands in different target classes.

Since targets are represented by the reference ligands, the TF predictive ability initially depends on the representatives and diversity of these ligands. Targets with well-characterized interaction profiles, particularly those with multiple known ligands, tend to yield more reliable predictions. This observation was confirmed by our findings, which demonstrated that targets with a larger number of reference ligands exhibited significantly improved performance in RE, as shown in Figure 9A. Specifically, when compared to targets with only ten reference ligands, those with more reference ligands showed an improvement in RE_1_, RE_5_, and RE_10_ of 0.17, 0.17, and 0.16, respectively. Researchers should prioritize targets with well-characterized interaction profiles and multiple known ligands for TF studies to maximize the prediction reliability. For the convenience of researchers, Figure 9B highlights a selection of targets with the largest number of reference ligands. Among these targets, kinases and membrane receptors (especially G protein-coupled receptors) constitute a substantial proportion, which could be because for most molecules, experimental studies have historically focused on a limited set of targets, such as kinases or membrane receptors (Entzeroth et al., 2009). Detailed statistical data on the number of ligands for all targets are provided in the Supplementary Material.

**FIGURE 10:**
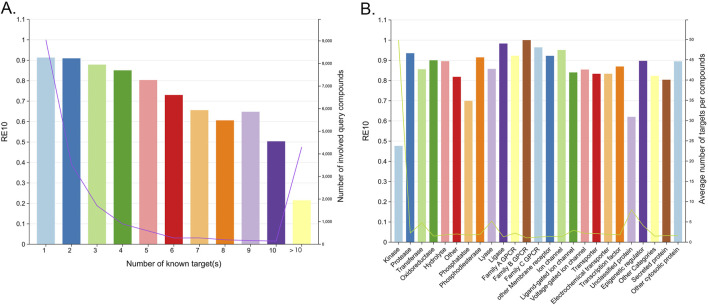
**(A)** RE_10_ for multiple baskets of the query compounds associated with different numbers of known targets. **(B)** RE_10_ for different target classes.

For molecules associated with multiple targets (corresponding to promiscuous query molecules (Peón et al., 2016)), the retrieval of all relevant targets was identified to be a daunting task. As shown in Figure 10A, the RE_10_ varied significantly, ranging from 91.32% to 50.35%, as the number of known targets for the query compounds increased from 1 to 10. Furthermore, target classes whose ligands are associated with a larger number of targets are more challenging to hit successfully. Statistically, kinases, which are known for their promiscuity, exhibited particularly low RE_10_ values below 0.5, as shown in Figure 10B. Despite these challenges, the similarity-centric TF models demonstrated notable performance. Given that an approved drug currently has an average of eight known targets (Peón et al., 2016), the fact that the RE_10_ of query compounds with 1 to 9 known targets exceeded 60% still highlighted the capability of the similarity-centric TF method, as shown in Figure 10A. These indicate that TF models can effectively enrich active targets for most compounds and potentially identify novel indications for existing drugs or compounds under investigation.

## 4 Discussion

Modern target-prediction tools augment drug discovery efforts in a range of applications by effectively narrowing down the set of potential targets to be validated in wet-lab experiments. In the present study, several baseline similarity-centric TF models were constructed with the aim of providing clues for enhancing the confidence of enriched hit targets.

The models applied in our study serve as the foundation for most state-of-the-art similarity-based TF models. Compared to tools such as SwissTargetPrediction (Daina et al., 2019) or several Polypharmacology Browser 2 (PPB2) models (Awale and Reymond, 2019), which typically employ similarity searching as an initial filter followed by machine learning models as a secondary filter, our models simulate the fundamental baseline of these multi-step methods. A proverb describes that “the underlying frame determines the superstructure.” If the foundational models are well-built, the final TF models will perform better and be more interpretable. Based on this new perspective, our study provides a lower bound for current similarity-centric models and offers essential insights and fundamental logic that can guide the development of the similarity-centric TF methods. Moreover, our baseline models have been validated to be robust, which demonstrates that our findings are reliable.

In the field of target prediction, 2D molecular fingerprints were widely used to represent chemical structures as they are easy to generate and fast to compare by using binary representations. Our study employed a variety of fingerprint types, including circular fingerprints, fingerprints based on molecular fragments/substructures, and fingerprints based on molecular topology/shape. The rigorous performance highlighted the robustness of the baseline models constructed using these 2D fingerprints. Among them, circular fingerprints, which provide a detailed description of molecular structures, emerged as being particularly effective and are recommended for application in TF models.

Our findings highlight similarity scores as a quantitative measure of reliability for enriched targets. The crucial role of similarity thresholds in enhancing the confidence of potential targets identified by similarity-centric models is emphasized. The applications of similarity thresholds can be considered the addition of inactive data, specifically, known non-targets for molecules, which are referred to as background noise in similarity-centric models. This approach helps reduce the false positive rates. Compared to machine learning TF models, similarity-centric TF models have the advantage of being effective even when the dataset of active compound–target interactions or inactive data is limited (Yang et al., 2020).

In practice, the evidence directly shows that for researchers using the similarity-centric models, if the similarity between the query compound and the nearest ligand of a target is below a predefined threshold, the target cannot be considered potential, regardless of its rank order. It is suggested that more flexible and confidential predictions can be obtained by automatically adjusting the threshold of similarity values in tools such as SwissTargetPrediction, PPB2, PPB, HitPickV2, TargetHunter, MolTarPred, and TarPred.

Our results also lay the groundwork for developing more robust and reliable target prediction models in the future. For example, several nearest reference ligands, similarity thresholds, fine fingerprints, and a more intensive reference library were suggested. By integrating these factors into models, researchers can make more informed decisions based on the predictions and prioritize targets for further investigation more effectively, ultimately accelerating the drug discovery process.

## 5 Conclusion

In summary, this study demonstrates that the confidence of similarity-based computational TF models can be enhanced by applying similarity thresholds and considering additional factors such as fingerprint selection, ensemble modeling, and the target–ligand interaction profiles. Notably, our study is the first to highlight similarity scores as a quantitative measure of reliability for enriched targets. Our findings propose the concept of similarity thresholds and identify effective similarity thresholds for different fingerprints to enhance the reliability. These novel insights provide a foundation for developing more robust TF models, thereby improving the efficiency and reliability of target identification in drug discovery.

## Data Availability

The original contributions presented in the study are included in the article/Supplementary Material; further inquiries can be directed to the corresponding author.
